# Hemoptysis: From Diagnosis to Treatment

**DOI:** 10.5334/jbsr.3378

**Published:** 2023-11-21

**Authors:** Antoine Khalil, Selma Attia, Ahmad Tibaoui, Aslam Souli, Ralph Khoury, Waqaas Mohammad

**Affiliations:** 1 CHU Bichat- Claude Bernard, APHP, FR

**Keywords:** Hemoptysis, lung cancer, Angio-CT, Bronchial artery embolization

## Abstract

Management of hemoptysis begins with an angio-CT to identify the location, the bleeding vessel, mapping of systemic arteries and the cause of the hemoptysis.

Endovascular treatment is the first-line therapy, in 90% of cases by embolization of the systemic arteries and in 10% of cases by occlusion of the pulmonary arteries.

## Indication

The recognized definition of life-threatening hemoptysis is a flow rate higher than 200mL/24h in a patient with normal respiratory function. This threshold can be decreased to 50–100 mL/24h in a patient with respiratory impairment and low respiratory reserve. The major risk of hemoptysis is asphyxia and, exceptionally, blood loss. Life-threatening hemoptysis is the main indication for embolization. However, repeated chronic hemoptysis may lead to bronchial artery embolization.

## Etiologies

**Figure 1:**
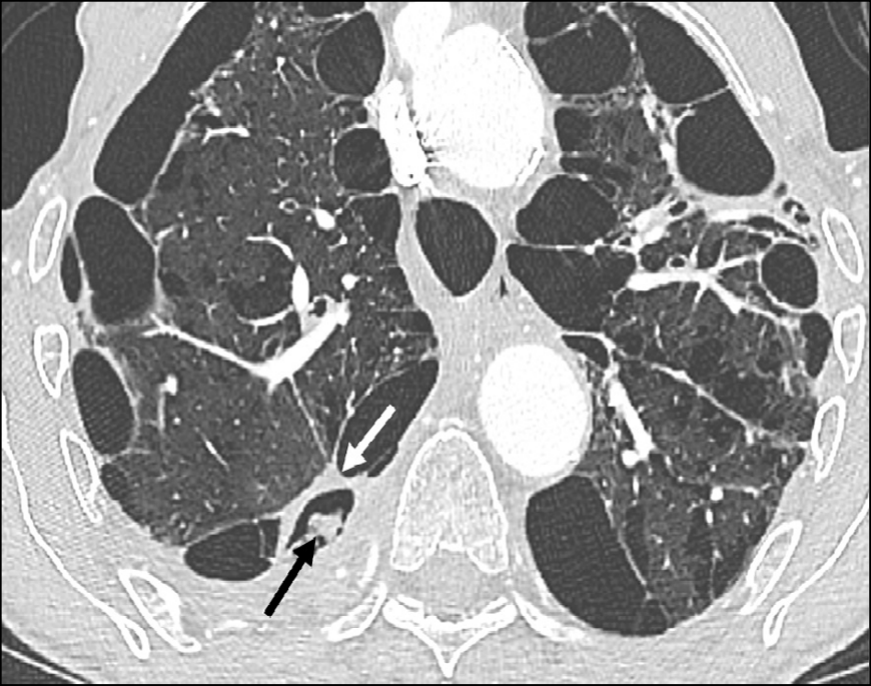
Axial CT section in the parenchymal window shows thickening (white arrow) of the wall of an emphysema bulla with endocavitary material (black arrow).

The five causes of hemoptysis requiring embolization, accounting for over 85% of hemoptysis cases, are: acute and sequelae of tuberculosis, bronchiectasis, aspergillosis (aspergilloma (Figure 1), chronic and acute aspergillosis), lung cancer, and idiopathic hemoptysis [1].

## Morphological workup

**Figure 2:**
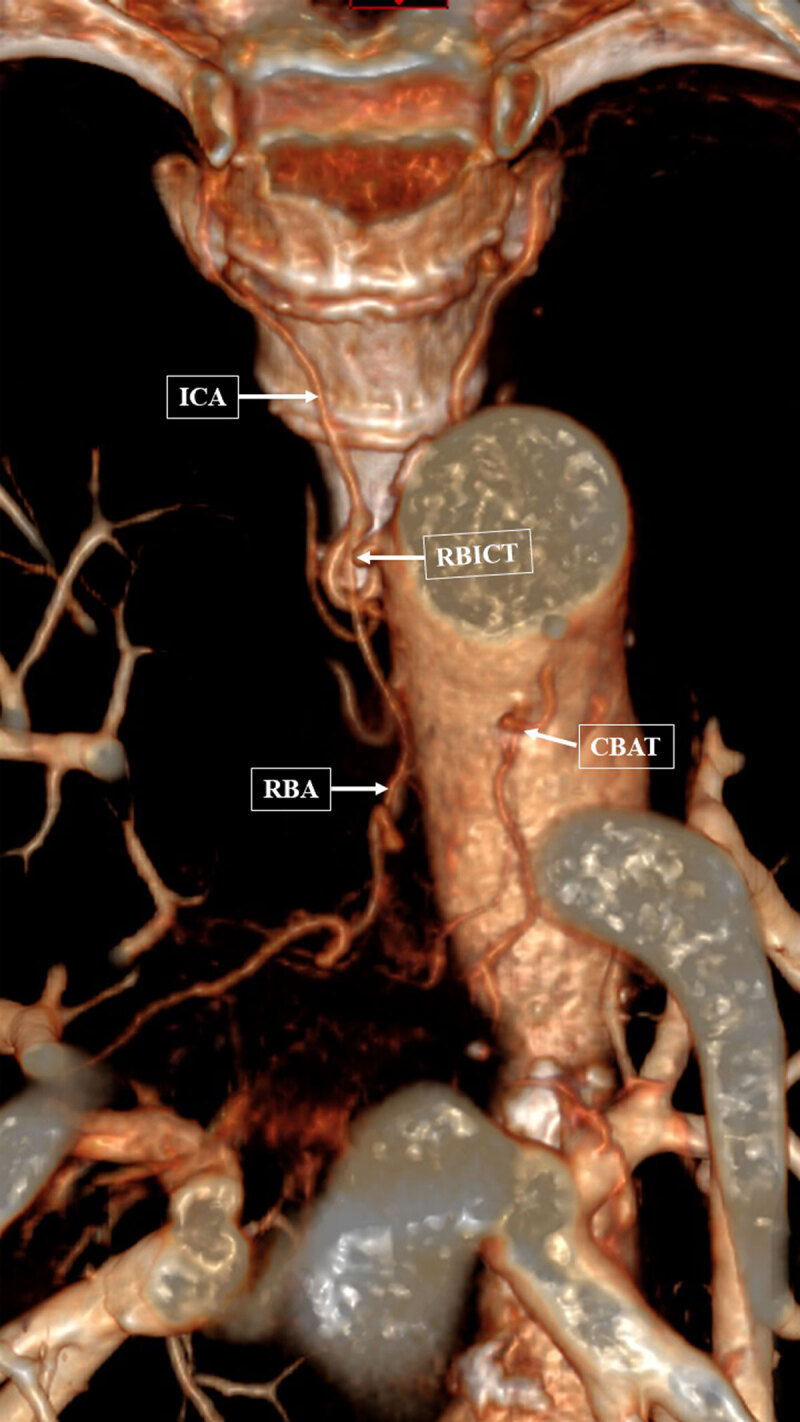
Volume-rendered technic reconstruction in the coronal plane shows an enlarged right bronchial intercostal trunk (RBICT) with its intercostal artery (ICA) and right bronchial artery (RBA). The common bronchial arterial trunk (CBAT) is also clearly visible.

**Figure 3:**
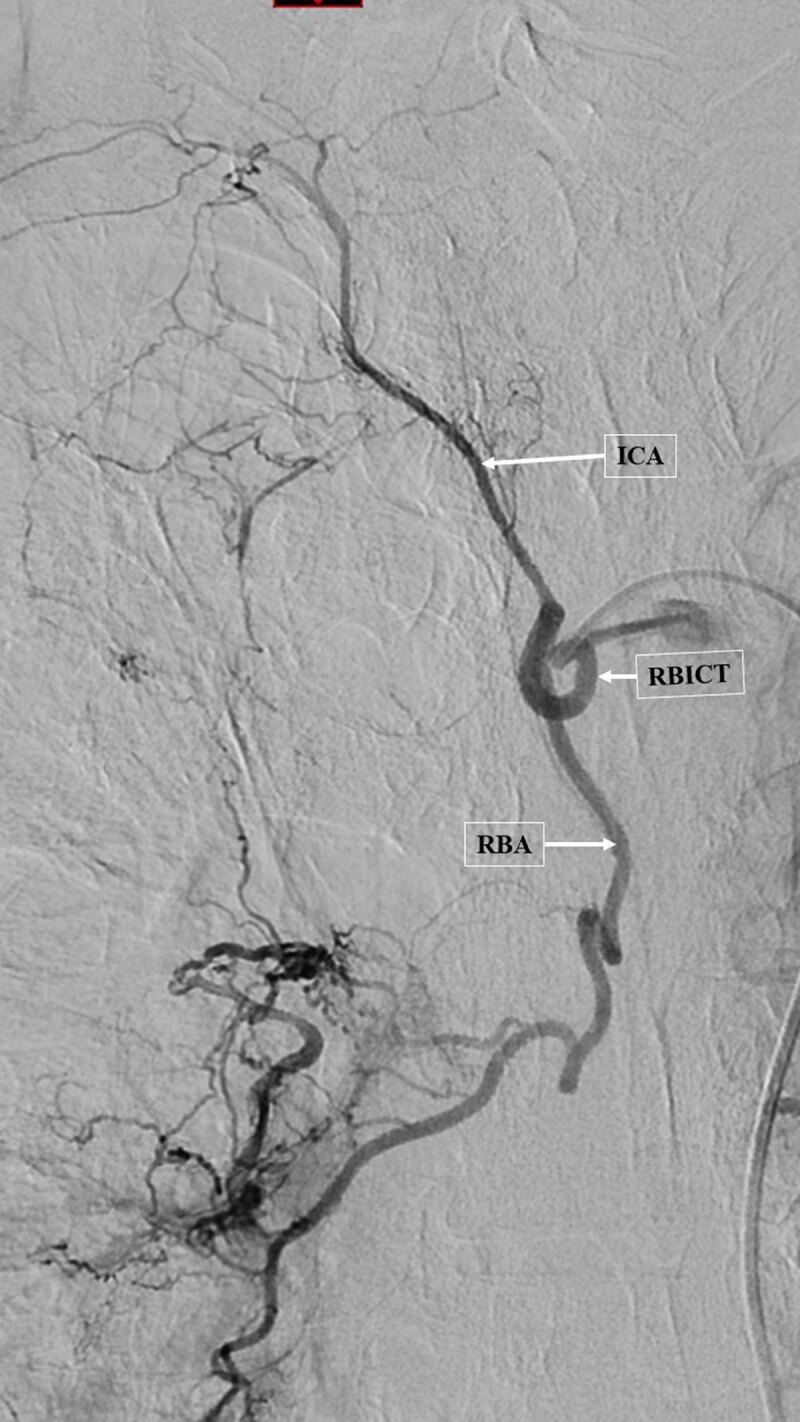
Angiogram of the enlarged right broncho-intercostal trunk (RBICT) showing the intercostal artery (ICA) without middle spinal cord artery and a right bronchial artery (RBA) with a systemic-pulmonary shunt related to systemic hypervascularization.

Chest angio-computed tomography (CT) with opacification of the entire thoracic vascular system (pulmonary arteries, pulmonary veins and thoracic aorta and its branches) is an essential examination in the initial assessment of hemoptysis. It helps to localize the bleeding site, identify the bleeding vessel, map the systemic bronchial (BSA) and non-bronchial arteries (NBSA) (Figure 2, 3) and, finally, identify the etiology [2, 3]. The indication for chest angio-CT must be wide enough to be performed even when the volume of hemoptysis is 20 to 30 mL.

Bleed localization is based on focal parenchymal abnormalities (‘condensation/ground-glass’ opacities); in the case of diffuse involvement, CT cannot localize the site of bleeding. In most cases, bleeding occurs in the systemic arteries (over 90%) or in the pulmonary arteries (less than 10%). Systemic involvement in bleeding is identified by excluding bleeding of pulmonary origin. In fact, bleeding from the pulmonary artery presents signs that are suggestive of the diagnosis. CT signs pointing to pulmonary arterial involvement are, in decreasing order of frequency, necrosis or cavitation, visualization of an irregular pulmonary artery in the wall of the necrosis, and identification of a false pulmonary arterial aneurysm.

## Embolization

The aim of embolization is to occlude the vessel or vessels involved in the bleeding. These vessels are the systemic arteries (BSA/NBSA) in the majority of cases with a femoral arterial approach, and in less than 10% of cases is the pulmonary artery with a femoral venous approach.

A 53-year-old man was treated for chronic cavitary aspergillosis complicating extensive emphysema. He was admitted emergently with 250 mL of hemoptysis in two episodes over the past 24 hours.

## Embolization materials

*Particles*. The size of the physiological shunt between the bronchial systemic arteries and the pulmonary arteries is around 250–300 microns. This shunt enlarges in inflammatory pathologies such as bronchial dilatation, aspergillosis, or tuberculosis and can exceed 1000 microns (1 mm). This means that the size of embolization particles must be greater than 400 microns, and adapted to the presence of enlarged shunts, otherwise the particles will pass through the shunt and cause iatrogenic pulmonary embolism and pulmonary infarction. *Coils*. The use of coils should be reserved for definitive occlusion distal to the pulmonary hilum. Anastomoses between systemic arteries in the mediastinum can cause reperfusion behind the occlusion, leading to persistent or early recurrence of hemoptysis. *Other*. Rarely, other materials may be used, such as liquid emboli or covered vascular stent.

## Results

The results of embolization vary from 70 to 90%, depending on the etiology.

## Recurrences

Causes of early recurrence include a different mechanism of bleeding or incomplete embolization, missing of one or more target arteries. Causes of late recurrence are repermeabilization of the vessels or progression of the disease that caused the bleeding.

## Complications

Major complications are mainly linked to embolization of non-target arteries (cerebral, digestive or median spinal cord artery, etc.).
